# 67409 Quantifying Unmeasured Confounding in Relationship between Treatment Intensity and Outcomes among Older Patients with Hodgkin Lymphoma (HL) using Surveillance, Epidemiology and End Results (SEER)-Medicare Data

**DOI:** 10.1017/cts.2021.531

**Published:** 2021-03-30

**Authors:** Angie Mae Rodday, Theresa Hahn, Peter K. Lindenauer, Susan K. Parsons

**Affiliations:** 1 Tufts Medical Center; 2 Roswell Park Comprehensive Cancer Center; 3 University of Massachusetts Medical School - Baystate

## Abstract

ABSTRACT IMPACT: E-values can help quantify the amount of unmeasured confounded necessary to fully explain away a relationship between treatment and outcomes in observational data. OBJECTIVES/GOALS: Older patients with HL have worse outcomes than younger patients, which may reflect treatment choice (e.g., fewer chemotherapy cycles). We studied the relationship between treatment intensity and 3-year overall survival (OS) in SEER-Medicare. We calculated an E-value to quantify the unmeasured confounding needed to explain away any relationship. METHODS/STUDY POPULATION: This retrospective cohort study of SEER-Medicare data from 1999-2016 included 1131 patients diagnosed with advanced stage HL at age ≥65 years. Treatment was categorized as: (1) full chemotherapy regimens (‘full regimen’, n=689); (2) partial chemotherapy regimen (‘partial regimen’, n=175); (3) single chemotherapy agent or radiotherapy (‘single agent/RT’, n=102), or (4) no treatment (n=165). A multivariable Cox regression model estimated the relationship between treatment and 3-year OS, adjusting for disease and patient factors. An E-value was computed to quantify the minimum strength of association that an unmeasured confounder would need to have with both the treatment and OS to completely explain away a significant association between treatment and OS based on the multivariable model. RESULTS/ANTICIPATED RESULTS: Results from the multivariable model found higher hazards of death for partial regimens (HR=1.81, 95% CI=1.43, 2.29), single agent/RT (HR=1.74, 95% CI=1.30, 2.34), or no treatment (HR=1.98, 95% CI=1.56, 2.552) compared to full regimens. We calculated an E-value for single agent/RT because it has the smallest HR of the treatment levels. The observed HR of 1.74 could be explained away by an unmeasured confounder that was associated with both treatment and OS with a HR of 2.29, above and beyond the measured confounders; the 95% CI could be moved to include the null by an unmeasured confounder that was associated with both the treatment and OS with a HR of 1.69. Of the measured confounders, B symptoms had the strongest relationship with treatment (HR=2.08) and OS (HR=1.38), which was below the E-value. DISCUSSION/SIGNIFICANCE OF FINDINGS: Patients with advanced stage HL who did not receive full chemotherapy regimens had worse 3-year OS, even after adjusting for potential confounders related to the patient and disease. The E-value analysis made explicit the amount of unmeasured confounding necessary to fully explain away the relationship between treatment and OS.

